# Case report: Combination of veno-arterial extracorporeal membrane oxygenation and intra-aortic balloon pump in a young male patient with refractory cardiogenic shock due to aluminum phosphide poisoning

**DOI:** 10.3389/fcvm.2023.1226827

**Published:** 2023-09-12

**Authors:** Oleg Dukhin, Danila Bala, Evgeny Felker, Polina Golovina, Mariya Tretyakova, Boris Haes, Polina Savvinova

**Affiliations:** ^1^ Cardiology Department, Moscow State University of Medicine and Dentistry, Moscow, Russia; ^2^ Moscow Department of Healthcare, Moscow Clinical City Hospital Named After I.V. Davydovsky, Moscow, Russia

**Keywords:** cardiogenic shock, VA-ECMO, aluminium phosphide, IABP, mechanical circulatory support

## Abstract

**Background:**

Acute toxic myocardial damage may be accompanied by the development of cardiogenic shock (CS), the mortality from which is still unacceptably high. Since there is no specific antidote for many types of toxins, treatment of such patients includes various measures of hemodynamic and respiratory support. The paper presents a case of refractory CS due to possible aluminum phosphide (AP) poisoning.

**Case summary:**

A 20-year-old man was admitted to the emergency department 4 days after home inhalation of AP due to complaints of nausea, vomiting, abdominal and chest pain. Over the next few hours, he rapidly developed CS, which was refractory to conservative treatment. Therefore, veno-arterial membrane oxygenation (VA-ECMO) was performed, during which hemodynamics stabilized, but later there were signs of left ventricular overload. To unload the left ventricle (LV), an intra-aortic balloon pump (IABP) was implanted, which significantly improved the patient's condition. After 6 days the patient was decannulated, and a few more days later IABP was discontinued. Subsequently, the patient was treated for sepsis due to bilateral pneumonia and acute respiratory distress syndrome and optimal medical therapy for heart failure was prescribed. The patient was discharged after 34th day of hospitalization.

## Introduction

Aluminum phosphide (AP) is a pesticide that is often used against insects and rodents in various developing countries (1). The interaction of AP with water produces phosphine, a toxic mitochondrial poison disrupting the processes of cellular respiration (2). AP poisoning manifests a wide range of symptoms, including gastrointestinal, cardiovascular, respiratory, renal and etc (3).

Mortality in AP poisoning ranges from 20%–60% (4), and in the case of cardiogenic shock (CS) it can reach 84.4% (5).

We present the clinical case of a 20-year-old man with accidental home exposure to AP resulting in CS due to acute biventricular heart failure, acute pneumonitis and acute respiratory-distress syndrome (ARDS), acute kidney injury (AKI), and subsequent sepsis. The patient was successfully maintained with extracorporeal membrane oxygenation (ECMO), followed by the intraaortic balloon pump (IABP) implantation due to left ventricle (LV) overload and several sessions of continuous renal replacement therapy (CRRT).

## Case presentation

**Figure 1:**
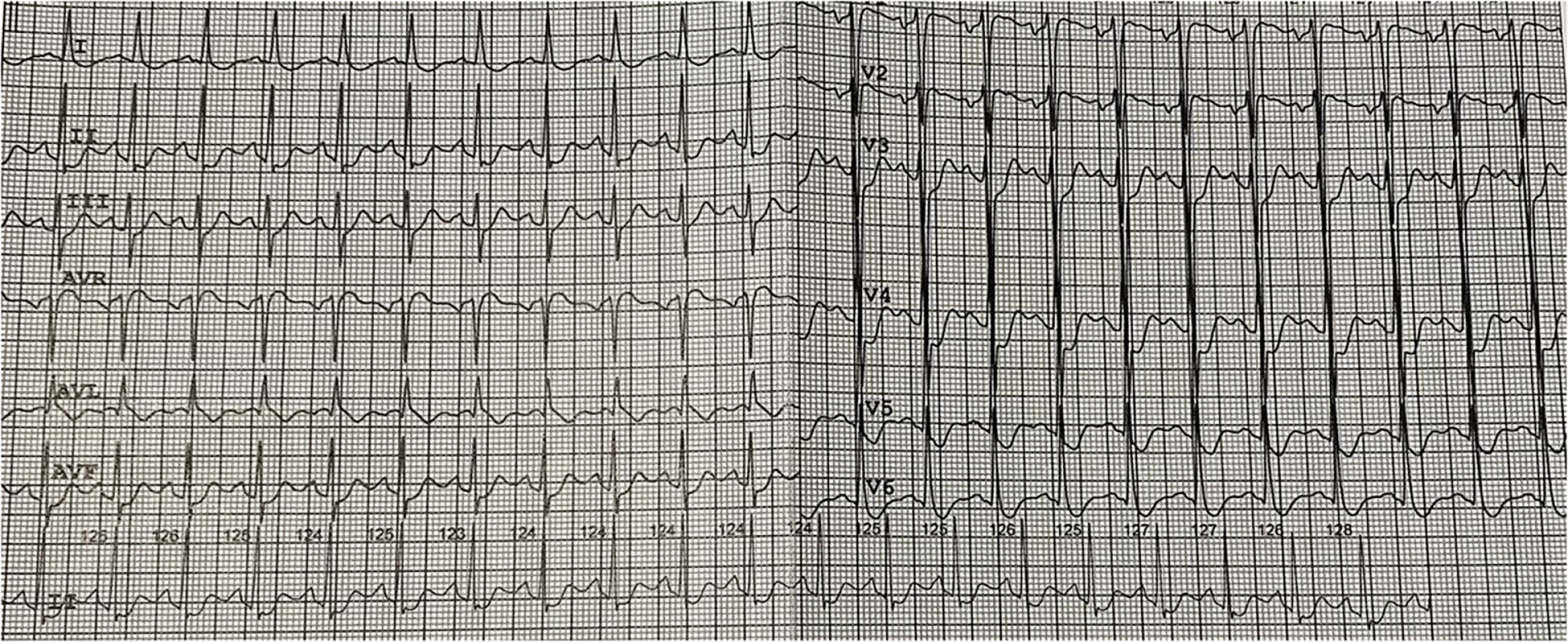
Electrocardiogram on admitting to the hospital.

The patient was a 20-year-old Asian male admitted to emergency room (ER) due to headache, nausea, vomiting, blood pressure elevation (up to 160/100 mm Hg), chest and abdominal pain. These complaints occurred within 4 days prior to hospitalization after AP cockroach baiting. The patient's medical history was unremarkable. During these 4 days, the patient self-treated at home (taking captopril, hydrochlorothiazide, furosemide, meglimine sodium succinate, activated charcoal). On admission, the patient's respiratory rate was 26/min, blood pressure was 100/60 mm Hg, heart rate 130/min. The patient was pale with diaphoresis. ECG showed sinus tachycardia with a heart rate of 130 per minute, ST depression in I, II, III, aVF, V3-V6 leads, ST elevation aVR, V1 (Figure 1).

Laboratory studies revealed leukocytosis (12.4 × 10^9^/L), increased level of troponin I (44,1 ng/L), procalcitonin (11,9 ng/ml), C-reactive protein (125,5 mg/L), total bilirubin (28,1 µ/L; free bilirubin 19,3 µmol/L), normal levels of transaminases (AST 35 IU/L; ALT 21 IU/L), creatinine (54 µmol/L), alpha-amylase (47 IU/L). Blood gas analysis revealed acute respiratory alkalosis [pH 7,5; pCO2 26 mm Hg; pO2 108 mm Hg; lactate 6,1 mmol/L; HCO3(−) 20,3 mmol/L; BE −0,5 mmol/L].

**Figure 2:**
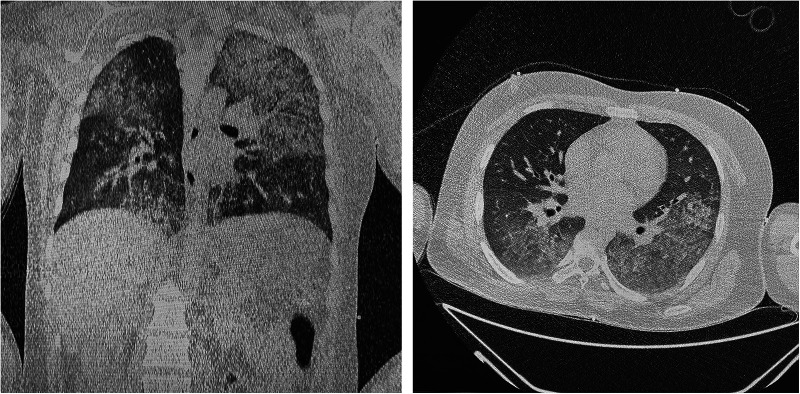
Chest CT-scan.

**Figure 3:**
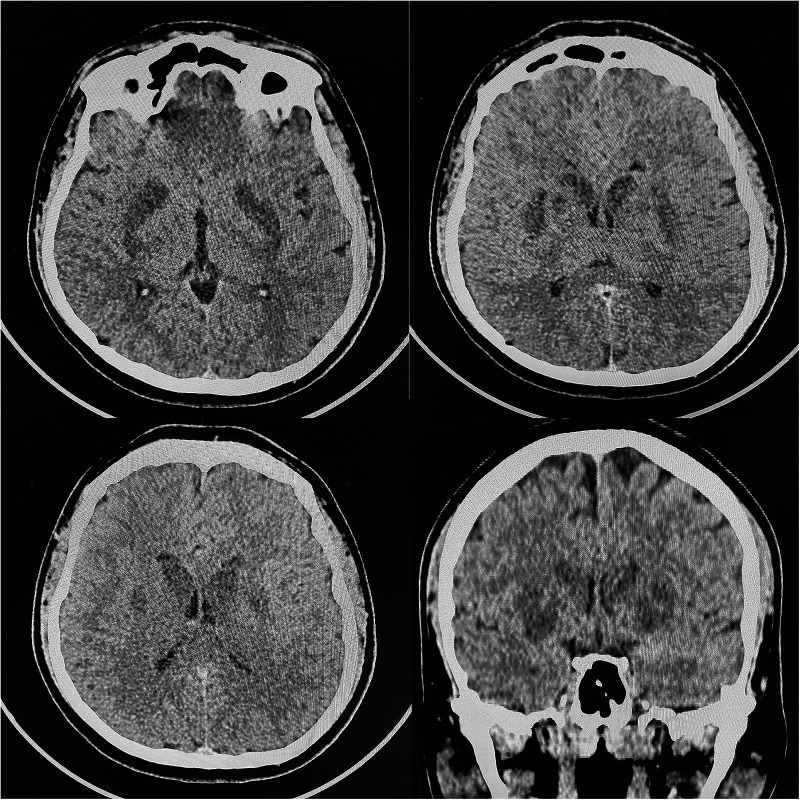
Head CT-scan.

Transthoracic echocardiography (TTE) demonstrated reduced left ventricle ejection fraction (LV EF) (24%–26%), diffuse hypokinesis of the LV, elevated systolic pulmonary artery pressure (47 mm Hg), moderate mitral regurgitation, normal right atrial size (right atrium area 10 cm^2^) and right ventricle end-diastolic size (31 mm) and a large number of B-lines in the lungs. Chest CT scan revealed a picture of polysegmental foci of ground glass opacities and signs of ARDS (Figure 2). PCR for SARS-CoV-2 was double negative. CT of the brain showed a picture of foci of decreased density in the basal ganglia and thickened contents in the anterior horns of the lateral ventricles (Figure 3). The blood, urine and hair test analysis for the content of metals, toxins and poisons was performed. The results of the toxicological analysis were negative (which was most likely due to the patient's admission to the hospital only several days after AP exposure).

Within a few hours after admission, the patient was lethargic and difficult to awaken, whereas the progression of respiratory failure was noted. In this regard, the patient was intubated and transferred to mechanical ventilation. A Swan-Ganz catheter was inserted for comprehensive hemodynamic profiling (right atrial pressure 22/13 mm Hg; right ventricle pressure 50/35 mm Hg; pulmonary artery pressure 46/31 mm Hg; pulmonary capillary wedge pressure 25 mm Hg; cardiac output 3,95 L/min).

For the purpose of detoxification, a session of renal replacement therapy [continuous veno-venous hemodiafiltration (CVVHDF)] was performed. Despite this, the patient's clinical condition did not improve, the levels of creatinine (53,0 µmol/L), C-reactive protein (132 mg/L) and lactate (7,0 mmol/L) remained unchanged). The patient's hemodynamics continued to deteriorate (blood pressure dropped to 60/30 mm Hg, anuria was observed), despite norepinephrine infusion in increasing doses (up to 0,8 mcg/kg/min) and adequate infusion therapy. Dobutamine was not used due to severe tachycardia. Besides, respiratory distress was observed. The negative dynamics in blood gas analysis was also recorded [pH 7,42; pCO2 44 mm Hg; pO2 75 mm Hg; lactate 7,0 mmol/L; HCO3(−) 28,5 mmol/L; BE 3,2 mmol/L]. TTE revealed worsening of LV EF to 20%, RV size and contractility remained unchanged. Due to the young age of the patient, the absence of significant risk factors and complaints of angina, the diffuse nature of the decrease in LV contractility according to TTE and the nonspecific nature of ECG changes, as well as the relationship of the clinical picture with AP exposure, the presence of an acute coronary event in this case was considered unlikely due to which it was decided to refrain from coronary angiography. Therefore, the patient was cannulated for peripheral veno-arterial extracorporeal membrane oxygenation (VA-ECMO) via femoral vessels. VA-ECMO support was started at flow 3 L/min. Subsequently there was a tendency to hemodynamic stabilization, but later there were noted signs of LV overload (LV dilation, elevated filling pressures and pulmonary edema). In order to unload the LV, an IABP was implanted (in 2:1 mode due to tachycardia). On the 5th day of ongoing therapy the stabilization of hemodynamics was observed. The ECMO weaning protocol was performed, and after 6 days the patient was decannulated from VA-ECMO and on the 10th day IABP was stopped. Due to persistent decrease in the LV EF and increase in the level of NT-proBNP (2,730 pg/ml), therapy with angiotensin receptor/neprilysin inhibitor (50 mg b.i.d) and bisoprolol (10 mg o.d.) was started.

In addition, during hospitalization patient developed sepsis due to polysegmental bilateral pneumonia. Empiric antibacterial therapy was carried out. Further antibacterial therapy was adjusted to the sensitivity (P. aeruginosa, S. aureus) to the combination of piperacillin-tazobactam, inhalated colistin, P. aeruginosa bacteriophage (Pseudomonas aeruginosa bacteriophage solution) (purified sterile filtrate of Pseudomonas aeruginosa phagolysate), 30 ml t.i.d. per os). Due to the development of AKI and sepsis the patient underwent two sessions of CVVHDF (oXiris, Baxter, USA). Subsequently, the patient's condition stabilized, normothermia and a normalisation in the level of inflammatory markers were noted.

**Figure 4:**
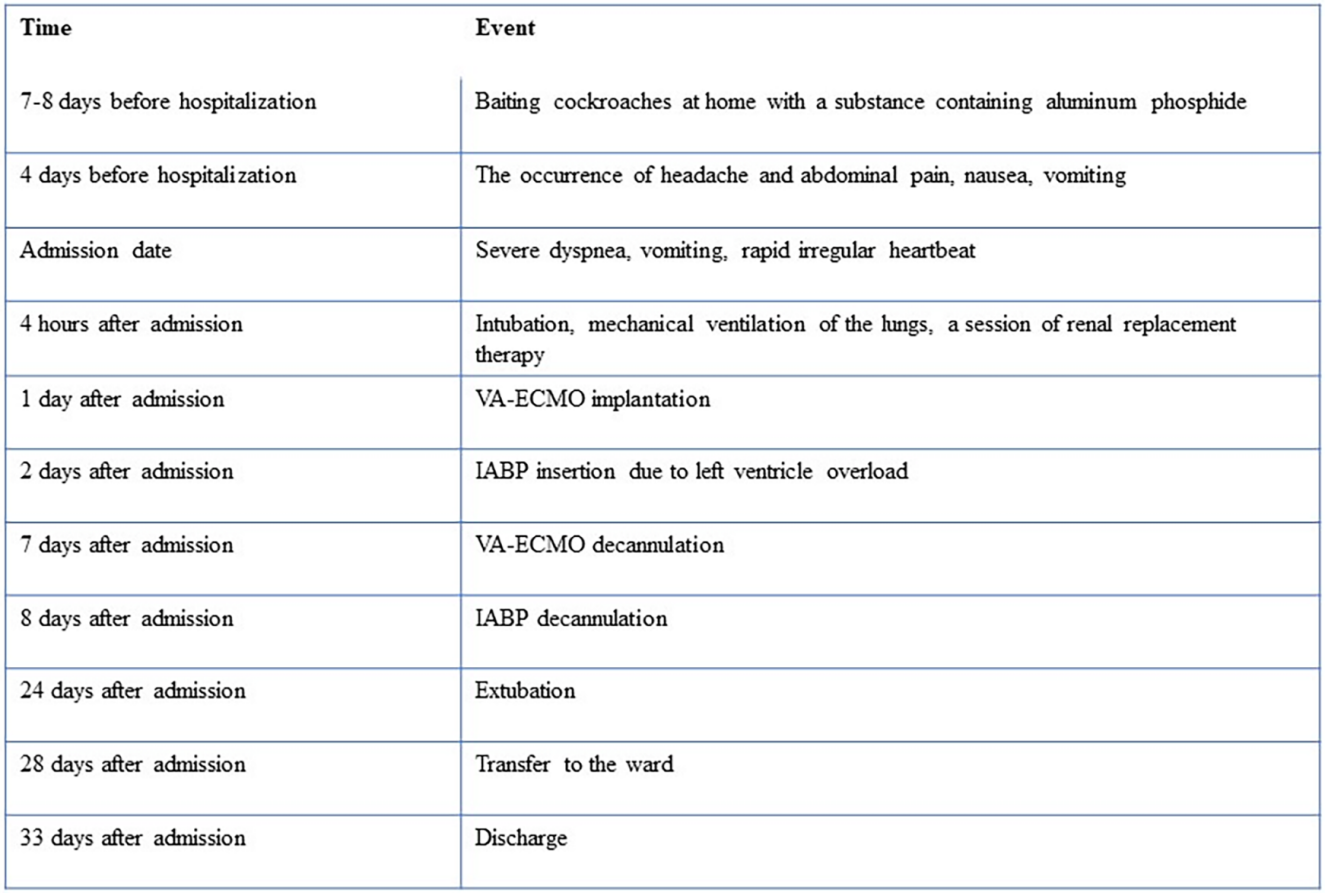
Case timeline.

The patient required a long-term rehabilitation due to neurological complications (pseudobulbar syndrome, impaired higher mental activity) and asthenia. During hospitalization, the patient underwent repeated echocardiography. According to the control study, on the 21st day of hospital stay, there was a positive trend in the form of an increase in the LV EF up to 50%, normalization of the end-diastolic volume of the left ventricle (90 ml), ECG was unremarkable. The patient was discharged after 34th day of hospitalization. The patient was advised to continue the prescribed heart failure therapy for three months, followed by a decision on the need for correction of therapy. The case timeline is presented in Figure 4.

## Discussion

This clinical case demonstrates the experience of combined use of VA-ECMO and IABP in a young patient with CS and ARDS. Myocardial damage caused by different toxic agents has been described previously (4). Based on the case history and the bilateral nature of the brain lesion on CT (6), the toxic genesis of acute myocardial injury in the presented clinical scenario is most likely related to the use of AP. Although there are several ways of verifying AP poisoning (e.g., silver nitrate test or chromatography), verification of a toxic agent 4–5 days after toxin exposure is extremely difficult, therefore the history of AP exposure and clinical symptoms remain the basis of diagnosis in most cases (7).

AP is a highly effective and cheap pesticide and rodenticide. The main mechanism of action of AP is inhibition of cytochrome C, disruption of electron transport along the mitochondrial respiratory chain and, as a result, disruption of cellular respiration. The main cause of death in this cohort of patients is development of CS due to acute myocardial toxic damage and fluid loss (8). The most common other cardiovascular symptoms associated with AP poisoning are heart failure (9), pericarditis, hypotension (10), various cardiac arrhythmias and conduction disturbances (11). Several possible therapeutic options (such as almond oil, N-acetylcysteine, N-omega-nitro-L-arginine methyl ester, vitamin C) have been previously proposed, but there is currently no proven effective antidote (8, 12). Due to the presence of an increase in the level of inflammatory markers, the presence of ARDS, corticosteroid therapy was not initiated. In this regard, various supportive measures are currently the basis of treatment as a possible bridge to recovery.

Due to the reversibility of myocardial and pulmonary damage during AP poisoning, VA-ECMO appears to be a promising option for patients with CS. Although there is evidence for the use of VA-ECMO in CS due to acute myocardial infarction (13, 14), there are currently no randomized trials demonstrating the effectiveness of this method in patients with CS and ARDS due to AP poisoning. The rationale for the use of VA-ECMO in these patients is the possibility of hemodynamic support and provision of adequate tissue perfusion and oxygenation (15). In addition, successful experience of using VA-ECMO in case of poisoning of other etiologies (carbon dioxide, methanol, various drugs, etc.) has been described (16).

Previously several clinical cases with successful use of VA-ECMO in these patients have been described (17–23). B. Mohan et al. (2016) in observational study demonstrated a lower mortality rate in patients treated with VA-ECMO compared to those receiving conventional treatment (33.3% vs. 86.7%, *p* < 0,01) (24).

One of the possible complications of VA-ECMO is LV overload. Previously, several different strategies for LV unloading have been proposed (25). J. Lehoux et al. (2018) demonstrated a successful experience of atrial septostomy in a 3-year old female with CS and AP poisoning treated with VA-ECMO (17). Interestingly, several successful cases of IABP in patients with CS due to AP poisoning have been reported (26–28). W. Elabbassi et al. (2013) used IABP implantation in a patient with AP poisoning, which did not improve the patient's condition, and therefore VA-ECMO was started with a good result (29). On the contrary, in our clinical case, mechanical circulatory support was initiated as VA-ECMO and was further supplemented by IABP due to LV overload which resulted in subsequent hemodynamic stabilization.

The use of CRRT has been previously described in acute AP poisoning by several groups (20, 30). On the first day of hospitalization, we also performed a session of CRRT for detoxication purposes, which, however, did not bring significant clinical and laboratory improvement. During further stay in the CCU due to the development of sepsis and AKI, CRRT was performed two more times. A notable fact is the positive result of using bacteriophage therapy, which is now experiencing a renaissance and is a promising option in conditions of multidrug-resistant bacteria (31).

This clinical case demonstrates the combined use of several measures of hemodynamic support in a young patient with refractory CS and ARDS. It should be noted that the young age of the patient and the absence of significant comorbidity were a significant prerequisite for recovery. Given the high prevalence of AP poisoning and its high mortality, further research is needed to find an antidote for this compound.

### Limitations

Due to technical reasons, endomyocardial biopsy and cardiac magnetic resonance imaging were not performed. Nevertheless, according to the recommendations of the European Society of Cardiology, this clinical case meets the criteria for clinically suspected myocarditis, which is most likely caused by AP exposure (4).

## Conclusion

Thus, this clinical case demonstrates the experience of combined use of VA-ECMO and IABP in a patient with cardiogenic shock and ARDS due to acute myocardial injury with possible AP poisoning. The combination of these two methods of mechanical circulatory support may be a promising strategy in the treatment of CS combined with ARDS in this cohort of patients.

## Data Availability

The original contributions presented in the study are included in the article/Supplementary Material, further inquiries can be directed to the corresponding author.
